# Eleven Primary Melanomas, Colon Cancer, and Atypical Nevi in the Same Patient: A Case Report and Literature Review

**DOI:** 10.1155/2016/3145986

**Published:** 2016-02-28

**Authors:** Lea Juul Nielsen, Lisbet Rosenkrantz Hölmich

**Affiliations:** ^1^Department of Plastic Surgery, Breast Surgery and Burns Treatment, Rigshospitalet, 2100 Copenhagen, Denmark; ^2^Department of Plastic Surgery, Herlev Hospital, 2730 Herlev, Denmark

## Abstract

*Background*. As the incidence of cutaneous malignant melanoma increases in the Caucasian population, an increasing population of melanoma survivors is at risk of developing multiple primary melanomas (MPM) as well as secondary primary cancers.* Objective*. To present a case of a patient with atypical nevi, 11 primary melanomas over 33 years, and colon cancer and to review the literature on multiple primary melanomas, atypical nevi, and correlation of nonmelanoma cancers.* Conclusion*. The literature indicates that patients with MPM are not uncommon, although 11 primary melanomas are rarely described, that patients with MPM may have a better survival than patients with single primary melanoma, that atypical nevi are a risk marker of not only melanoma in general but also MPM, and that melanoma patients have a significantly increased risk of developing nonmelanoma skin and other cancers, which may be even higher for patients with MPM.

## 1. Introduction

The incidence of cutaneous malignant melanoma has been rapidly increasing in Caucasian populations worldwide for decades [1–7]. As public information, surveillance, and treatment improve, the survival rates improve also, leading to an increased population of long-term survivors after melanoma [3, 5–8]. Consequently, an increasing population of melanoma survivors is at risk of developing multiple primary melanomas.

The below presented case inspired a literature search and a narrative review on the subjects of multiple primary melanoma, atypical nevi, and correlation of melanomas and secondary primary cancers.

## 2. Case

**Figure 1:**
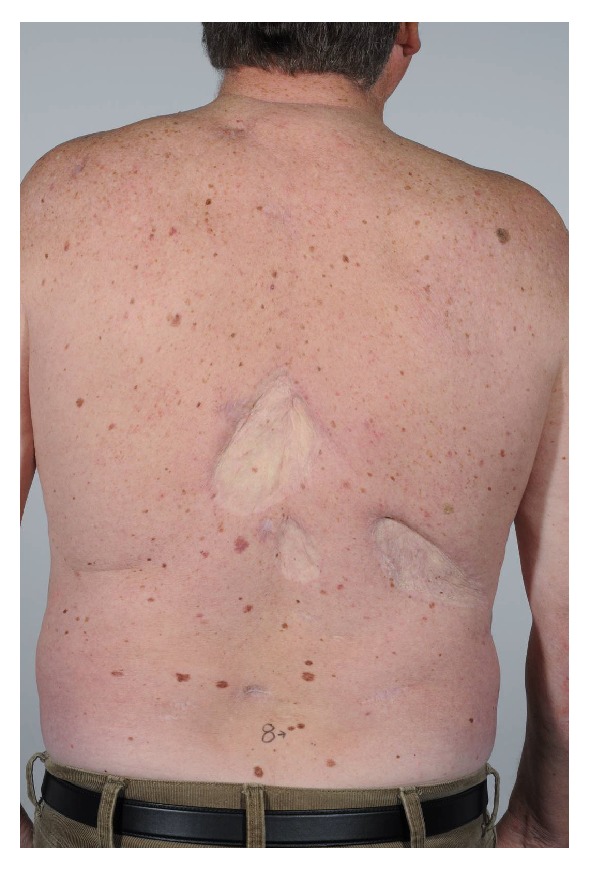
Patient with 11 melanomas over 32 years, showing areas with skin grafts due to previous treatment for melanoma as well as multiple dysplastic nevi.

A 70-year-old male, with no family history of melanoma, but with multiple atypical nevi, came for off-protocol follow-up in September 2013 (Figure 1). The patient first presented in 1981, at the age of 37, with malignant melanoma on the lower back and the following year another two primary melanomas were diagnosed on the lower back. Unfortunately, no histology on these melanomas is on record, but they are described in old case files. The patient had a 19-year period without recurrence or new tumours but presented again in 2001 with SSMM on the back, level 4, Breslow depth of 1 mm, and negative sentinel node biopsy. Over the next 12 years, the patient was followed up according to the protocols of the time and diagnosed with seven new primary melanomas, four invasive and three in situ melanomas (Table 1). In 2013, the diagnosis of a thick melanoma on the back required sentinel node biopsy. However, the lymphoscintigraphy did not demonstrate any hot spots and sentinel node biopsy could not be performed. The patient underwent a PET-CT-scan instead, which showed suspicious areas in the colon and a slightly enlarged spleen. Additional work-up did not explain the slightly enlarged spleen, but an adenocarcinoma of the sigmoideum was diagnosed and operated. The patient was genetically tested but found not to be carrier of either of the two high-risk genes CDK4 and CDKN2A.

## 3. Material and Method

A literature search on the 3 subjects, multiple primary melanomas, dysplastic nevi, and secondary malignancy in melanoma patients, was conducted from January 2014 until August 2014 using PUBMED and search terms “multiple malignant melanoma”, “Dysplastic Nevi”, “Atypical nevi”, “Dysplastic nevi syndrome AND multiple malignant melanoma”, “genetics AND multiple malignant melanoma”, and “multiple malignant melanoma AND subsequent cancer”.

Reference lists of included articles served to identify further sources.

## 4. Multiple Primary Melanomas

Pack et al. first described multiple primary melanomas (MPM) in 1952 in a report of 16 cases [9]. It has since been well established that 1.2%–12.7% of melanoma patients are at risk of developing one or more subsequent primary melanomas (Table 2). The risk of developing further primary melanomas appears to decrease drastically after the first two melanomas and for each subsequent melanoma as shown in Table 3.

The risk of developing subsequent melanomas appears to be highest the first year after diagnosis and decrease over time but is still elevated after 20 years compared to the background populations risk of a first melanoma [1, 10]. Hwa et al. showed an accumulated risk of developing a second melanoma within the first year of diagnosis of 4,1% and a third melanoma within the first year of diagnosis of the second melanoma of 26,7% [2].

Ferrone et al. found that 59% of patients with MPM developed the second primary melanoma within the first year [3] and Vecchiato et al. found that 36% of 194 patients were diagnosed with further melanomas within the first year; however, 26% of these actually presented with synchronous tumours at the time of debut [1].

The high risk of diagnosis of another melanoma within the first year is probably due to synchronous tumours but also due to increased surveillance.

### 4.1. Pathology of Subsequent Melanomas

Several studies demonstrate that the initial melanoma in patients with MPM usually is the thickest and that subsequent primary melanomas are not only significantly thinner than index lesions, but also less mitotically active and more rarely ulcerated [1–5, 10–14].

This is likely due to increased surveillance and earlier diagnosis [2–4, 10, 15], although theories of immune modulation [2, 14, 16–18] and less aggressive phenotypes have been proposed as well [2, 3, 5].

#### 4.1.1. Increased Surveillance

Patients diagnosed with melanoma are usually enrolled in a follow-up program where the skin is examined for recurrence, subcutaneous metastases, and new lesions suspicious of melanoma. Patients are also instructed to perform self-examination of the skin and seek medical attention in case of nevi changing, itching, or bleeding. This is likely to lead to earlier diagnosis of subsequent melanomas.

Supporting this theory is the finding that the second lesion is more often an in situ melanoma as well as the abovementioned findings that subsequent melanomas are significantly thinner. Murali et al., 2012, found that subsequent melanomas were significantly thinner and less mitotically active [10]. Interestingly, the subsequent melanomas, diagnosed more than three years after the first melanoma, when the patients were followed up less frequently, were thicker, more often nodular, and more often mitotically active than when the subsequent melanomas were diagnosed earlier, when the patients were followed up more frequently [10].

De Giorgi et al., 2010, found that patients with MPM who did not attend regular follow-ups had thicker subsequent melanomas than patients with MPM who did attend regular follow-ups [15].

#### 4.1.2. Immunization Effect

Various types of antigens are expressed in melanomas and can be recognized by cytotoxic T-lymphocytes, causing destruction of the tumour cells (tumour regression). Some have speculated that recognition of melanocytic differentiation antigens by the immune system could lead to the destruction of tumours in successive melanomas, manifesting as histologic regression [16]. Thus, tumour regression becomes suggestive of an immunization process.

Martín et al., 2014, reviewed 19 patients with MPM and found that the regression was significantly higher in successive melanomas than in the first tumour, suggesting an immunization effect from the first melanoma. Also, metastasis only occurred in patients without regression in their primary melanoma, suggesting a protective effect by regression [17].

In a study of 3676 patients, of which 1210 had MPM, by Murali et al., it was also found that regression was more common in subsequent melanomas than in the first [10].

Saleh et al., 2001, examined 23 patients with MPM and showed a significantly increased rate of regression when compared with controls with SPM. They also found a significantly improved survival in patients with MPM, even when controlling for other risk factors [16].

However, Zoller et al., 2010, examined 18 patients with MPM and found no significantly increased rate of regression comparing the first and the second primary melanoma [18].

#### 4.1.3. Phenotype

It has been proposed that a different phenotype in MPM versus single primary melanoma (SPM), causing a less aggressive tumour biology, leads to thinner subsequent melanomas and better prognosis [2, 3, 5]. Ferrone et al. support this theory and explain the difference in disease specific death between patients with SPM (15,8%) and MPM (5,6%) by a less aggressive phenotype [3], as do Bower et al. who found a better overall survival for patients with MPM compared to patients with SPM [5]. On the other hand, the finding by Hwa et al. that there was no significant difference in mitotic rate between SPM and MPM indicates that the tumour phenotype does not differ in terms of biologic aggression [2].

In conclusion, the studies are rather small and the sparse results are somewhat contradictory and offer no definite explanation to the finding that subsequent primary melanomas are thinner, less mitotically active, and more rarely ulcerated than index lesions. One explanation could in fact be that the causes are multifactorial; another is that the study groups vary greatly in age range, life style, follow-up period, and so forth and that the studies vary in design. Supporting the immunization theory however is that one of the significant advances in nonsurgical treatment of melanoma is the development of immune-boosting therapies, such as the monoclonal antibody, ipilimumab, which blocks cytotoxic T-lymphocytes antigen 4 (CTLA-4).

### 4.2. Prognosis

Kricker et al., 2013, examined a cohort of 2372 patients with SPM and 1206 patients with MPM and found that the main determinant of fatality was melanoma thickness (hazard ratio for melanoma > 4 mm was 7.68; 95% CI 4.46–13.23). Other independent predictors were ulceration, mitosis, and scalp location as well as increasing age. Adjusting for these factors, there was little difference in fatality between MPM and SPM. Thicker SPM, however, had a higher fatality than thicker MPM, indicating a difference in outcome between the two groups of patients related to factors other than closer surveillance and earlier diagnosis [19].

Several studies report a better prognosis for patients with MPM than those with SPM as shown in Table 4 [3–5, 14, 16, 19] However, one recent study including 878 SPM and 190 MPM patients from a population based registry reported on an increased risk of death from melanoma for patients with MPM compared to patients with SPM. Advanced statistics were applied, and different scenarios for determining the index tumour were tested; all found increased risk of death among MPM patients, and when considering the last invasive melanoma as the index melanoma, the HR of death was 2.76 (95% CI 1.20–6.32). Similar Breslow thickness was found in the first melanomas in the MPM and SPM group; however the thickest lesions were found in the MPM group. Adjusting for Breslow thickness did not eliminate the increased risk of dying and the authors could therefore not determine if the increased risk could be due to the multiple melanomas, increased thickness, or perhaps certain phenotype characteristic among MPM patients. It should be noted that information about ulceration was only available for about 20% of tumours which could have biased the results [20].

Savoia et al., 2012, reported on a cohort of 4938 melanoma patients, 270 with MPM, and showed a significantly improved overall survival as well as a better disease-free survival for patients with more than one primary melanoma. This result is attributed to the earlier diagnosis due to regular follow-up [4]. Hwa et al., 2012, found a better prognosis for patients with MPM, but no significant difference in mitotic activity, hence concluding that the better prognosis must be due to the increased surveillance rather than difference in phenotype [2].

Doubrovsky and Menzies, 2003, also reported on improved survival for patients with MPM; specifically, patients with three or more primary melanomas survived longer than anticipated [14].

It is important to bear in mind that these data are easily biased. Patients with a single primary lesion with a poor prognosis may not live long enough to develop subsequent melanomas.

Doubrovsky and Menzies tried to avoid this bias by adjusting for significant variables (age at diagnosis, Breslow depth, ulceration, and localisation of the melanoma). The estimated survival was calculated and survival bias was controlled by only using the data of patients diagnosed with MPM within 2 years after the primary diagnosis, but still the survival was better than expected for patients with > 3 melanomas [14].

### 4.3. Risk Factors for MPM

The occurrence of multiple primary melanomas is associated with family history, dysplastic nevus syndrome, and the germline CDKN2A [3, 10, 21–24]. Dysplastic nevus syndrome is discussed in Atypical Nevi.

In patients with one melanoma, a family history of melanoma confers an increased risk of subsequent melanomas [3, 23, 25, 26]. Ferrone et al. reported on 4484 melanoma patients of which 385 had MPM. 21% of patients with MPM had a family history of melanoma compared with only 12% of patients with SPM [3]. A large Swedish database study from 2014 found a generally increased risk of MPM with a family history of melanoma, similar for in situ and invasive melanoma. Interestingly, the risk was similar for two and three melanomas in one first-degree relative (FDR) and for one melanoma in two or more FDRs but was 10-fold elevated for four or more melanomas in one FDR and for two or more melanomas in two or more FDRs, indicating that this risk is genetically driven, especially for MPM [26].

Two high-risk genes associated with melanoma have been identified so far: CDKN2A and CDK4. These are only present in about 2% of a population of melanoma patients, but for melanoma patients with a family history of melanoma 20% to 60% are carriers of mutations in either CDKN2A or CDK4 [21]. For patients with MPM there is an increased prevalence of CDKN2A, mostly correlated with family history, but also independently. However, the studies showing a higher independent prevalence of mutations in the CDKN2A gene for patients with melanomas are small [21, 24].

## 5. Atypical Nevi

Clark Jr. and colleagues first introduced the term dysplastic nevi in 1978 [27]. Several names have since been used: B-K mole, Clark nevi, atypical nevi, dysplastic nevi, melanocytic atypia, and so forth. The International Agency for Research on Cancer (IARC) recommended using the term “atypical nevi” in 1990, but the term dysplastic nevi is still widely used in the international literature. Just as the name is a source of controversy, so is the exact definition. In 1990 the IARC recommended the following criteria for dysplastic nevi: a macular component in at least one area; in addition, presence of at least three of the following features: (a) border not well defined, (b) size 5 mm or more, (c) variegated colour, (d) uneven contour, and (e) presence of erythema [28, 29].

Many pathologists classify the dysplasia as mild, moderate, or severe, but little consensus prevails on the exact definitions and interobserver reliability has been poor in studies. Nonetheless, the degree of atypia has been shown to correlate with the risk of melanoma; moderate and severe atypia have been associated with an increased risk of melanoma, as discussed below [30].

Atypical nevi are found in 2–53% of the general population, depending on whether the diagnosis is clinical or histological [29, 31, 32]. A more accurate estimate for a Caucasian population is believed to be within 2–8% [28, 29, 31]. Atypical nevi are more frequent in people with skin types 1 and 2 than in people with skin types 3 and 4 [31]. They are most commonly located on the trunk but may occur at any anatomic site [30, 31]. Evidence suggests that sun exposure, in addition to genetic susceptibility, can affect the development of atypical nevi [31].

The presence of atypical nevi can appear in families as dysplastic nevus syndrome, characterized by multiple atypical moles that continue to appear in adulthood within a family setting [28], or more commonly, sporadically, in which a patient has a variable number of atypical nevi and no family history [30].

### 5.1. Atypical Nevi and Melanoma

Patients with atypical nevi have an increased risk of malignant melanoma, perhaps as high as 10-fold [28, 30].

The incidence of atypical nevi within a population of patients with melanoma is 18%–59% [3, 31] whereas the incidence for patients with MPM is slightly higher at 38%–63% [1, 3, 10, 12], suggesting that atypical nevi are markers of risk of additional melanomas. Ferrone et al., 2005, reported that, of 385 patients with MPM, 38% had atypical nevi compared with 18% of patients with SPM (*p* < 0,001) [3].

It is greatly discussed whether atypical nevi themselves are at risk of transforming into melanoma or if they should only be regarded as a risk marker [10, 28–34]. The incidence rate of melanomas arising in association with an atypical nevus is reported to be as low as 0,5% and as high as 46%, but most authors claim that it is important to recognize that most atypical nevi are benign and remain stable over time [31]. Murali et al., 2012, found that a higher proportion of subsequent than first melanomas in patients with MPM were associated with remnants of a contiguous dysplastic nevus (24% versus 15%), suggesting a higher risk of transformation from atypical nevi to melanoma in patients with MPM [10].

Tucker et al., 2002, followed 33 families with melanoma and atypical nevi and found that most atypical nevi were either stable over time or disappeared [34].

Hocker et al., 2013, reported on a cohort of 115 patients with atypical nevi. Clinically atypical nevi were nonradically removed (by puncture biopsy, curettage, or excision in close proximity) to confirm the histological diagnosis of AN. The patients were followed up for more than 17 years (up to 30 years) and none developed MM [33].

Reddy et al., 2013, investigated the reliability of biopsy: 580 atypical nevi were curettaged and then excised. All initial diagnoses of mild-to-moderate dysplasia were confirmed at excision biopsy, whereas 4% of the initially diagnosed moderate-to-severe dysplasia was changed to invasive melanoma on excision biopsy, concluding that biopsied nevi with mild-to-moderate dysplasia can be observed whereas nevi with moderate-to-severe dysplasia should be radically removed [32].

## 6. Risk of Developing Secondary Primary Cancer

As the number of melanoma survivors increases, a larger proportion will be at risk of developing not only a second melanoma, but also a second primary malignancy at another site.

Several studies confirm an increased risk of developing subsequent cancers, after a diagnosis of melanoma. The risk is estimated to be within 6–16% [5–8, 11, 35, 36]. The risk is highest within the first year of diagnosis of melanoma [8, 11] and is significantly higher for people with MPM compared to people with SPM [6, 8, 36].

Caini et al., 2014, conducted a meta-analysis, including 23 studies and more than 350.000 melanoma patients. They found that the risk of a second primary cancer was increased overall, although the summary relative risk (SRR) was only statistically significant when including subsequent melanomas as second primary cancers. Significantly increased SRR emerged for cancer in bone and cartilage (SRR 2.09), nonmelanoma skin cancer (NMSC) (SRR 4.01), soft tissue (SRR 6.8), colorectal (SRR 1.12), female breast (SRR 1.14), prostate (SRR 1.25), kidney (SRR 1.34). and non-Hodgkin lymphoma (SRR 1.37). Significantly reduced SRR was observed for cancer of the larynx (SRR 0.65) and cervix uteri (SRR 0.73) [8].

Jung et al., 2014, not included in Caini et al.'s meta-analysis, examined the risk of subsequent primary malignancies following both NMSC and melanoma but found results consistent with the above for 6884 melanoma patients, although no decreased risk of larynx and cervix cancer was found. They, on the other hand, reported on a markedly increased probability of developing a second primary cancer in patients under the age of 40, mostly contributed to secondary cutaneous malignancies [35].

Several hypotheses have been proposed for this association: One hypothesis is the exposure of simultaneously occurring risk factors; for example, melanoma is more frequent amongst high socioeconomic populations, as is breast and prostate cancer, whereas the reverse is seen for cancer of the larynx. The major risk factor for melanoma, exposure to solar radiation, is shared with patients with NMSC, but inversely associated with cancers like colorectal, prostate, and breast cancer. Other risk factors such as viruses (HPV) and immunosuppression are also discussed [6, 35, 36].

A second hypothesis is the follow-up and investigation theory: Patients diagnosed with melanoma are frequently examined by a physician and imaging and laboratory examinations are performed, corresponding to the finding that the risk of a second primary cancer is highest the first years after a melanoma diagnosis [7, 35]. A third hypothesis is the possible similarity in cellular pathogenic pathways and for instance both mutations in CDKNA2 and in BRAF are reported in other cancers, such as pancreatic and colorectal cancers [6, 7, 11, 35, 36].

These findings have considerable implications in terms of follow-up, urging an awareness of the patients' other symptoms, and possibly suggesting further diagnostic tests.

## 7. Conclusion

After reviewing the literature on the subject of MPM, it is found that patients with 11 primary melanomas are rare but that patients with MPM are not uncommon. The patient in the presented case has survived more than 33 years without metastases, which corresponds with the literature, of which the majority of studies indicate that patients with MPM may have a better survival than patients with SPM. The subject is, however, contradictory.

The patient has multiple atypical nevi, which are known to be a risk marker of not only melanoma in general but also MPM. The patient has no reported family history of melanoma and is not carrier of the high-risk genes CDKN2A and CDK4.

The patient was recently diagnosed with colon cancer, which also corresponds with the literature, reporting a significantly increased risk of developing a second primary cancer for patients with melanoma. This risk may be even higher for patients with MPM.

Long-term follow-up with full-body skin examination is mandatory for patients with high risk of melanoma to promptly diagnose not only disease progression but also new primary tumours. For patients with melanoma and atypical nevi, lifelong follow-up is recommended, either in a specialised melanoma unit or in close collaboration with the patient's general practitioner, with the use of clinical photos as an aid in diagnosing new melanomas, dermatoscopic monitoring, excision biopsy of suspected lesions, and increased attention towards other potential malignancies.

## Figures and Tables

**Table 1:** List of melanomas in the reported case.

Date	Localisation	Histology	Treatment
1981	Lower back	MM	Excision^*∗*^ + skin graft

1982	Lower back	MM	Excision^*∗*^ + skin graft

1982	Lower back	MM	Excision^*∗*^ + skin graft

Dec 2001	Back, right side	SSMM, 1 mm, level 4, negative SN.	Excision, 2 cm + SN biopsy from right ingvinae + right axilla.

Jan 2004	Back, right side	SSMM, 0.69 mm, level 2, no ulceration.	Excision, 1 cm

Oct 2006	Left shoulder	SSMM, 0.49 mm, level 3, no ulceration.	Excision, 1 cm

Oct 2006	Left thorax	SSMM in situ	Excision, 0.5 cm

March 2012	Left shoulder	SSMM, 0.78 mm, level 3, no ulceration, regression or mitoses.	Excision, 1 cm

Sept 2013	Lower back	SSMM, 1.83 mm, level 4, no ulceration, regression or mitoses.	Excision, 2 cm + skin graft, not possible to locate SN.

Sept 2013	Upper back	SSMM in situ, no ulceration or regression.	Excision, 0.5 cm

Sept 2013	Upper back	SSMM in situ, no ulceration or regression.	Excision, 0.5 cm

^*∗*^Margin not known.

**Table 2:** Studies with more than one melanoma.

Study	Number of study subjects	Country	Proportion (%) with more than one melanoma	Median follow-up time (years)
Martín et al. 2014 [17]	741	Spain	2.56	Not known
Tóth et al. 2013 [36]	740	Hungary	6	2
Hwa et al. 2012 [2]	788	USA	7.7	3.7
Vecchiato 2012 [1]	2987	Italy	7.2	4.8
Savoia et al. 2012 [4]	4938	Italy	6.4	Not known
Bower et al. 2010 [5]	2506	USA	1.9	5.5
De Giorgi et al. 2010 [15]	672	Italy	5.95	Not known
Uliasz and Lebwohl 2007 [13]	877	USA	12.7	Not known
Ferrone et al. 2005 [3]	4484	USA	8.6	2.2
Doubrovsky and Menzies 2003 [14]	5250	Australia	5.7	Not known
Burden 1994 [23]	3818	Scotland	1.2	Not known
Slingluff Jr. et al. 1993 [25]	7816	USA	3.6	4.8

**Table 3:** Studies with several primary melanomas.

Study	Number of patients with MPM	Proportion with 2 MM (%)	Proportion with 3 MM (%)	Proportion with 4 MM (%)	Proportion with ≥5 MM (%)
Moscarella 2013 [37]	71	79	17	1	3
Tóth et al. 2013 [36]	44	72.7	22.7	2.3	2.3
Hwa et al. 2012 [2]	61	72	13	8	7
Vecchiato 2012 [1]	210	79.4	12.4	6.2	1
Savoia et al. 2012 [4]	270	76.7	16.7	3.7	2.9
Ferrone et al. 2005 [3]	385	78	15	5	2
Doubrovsky and Menzies 2003 [14]	298	88.6	8.7	2.7	
Slingluff Jr. et al. 1993 [25]	283	82	11	3	

**Table 4:** Survival of patients with single or multiple primary melanomas.

Study	Number of patients SPM/MPM	Overall survival (OS) or mortality rate (MR) (5 y) SPM/MPM	Conclusion
Rowe et al. 2015 [20]	1068/190	3.0%/6.8% (*p* = 0.01) (melanoma specific mortality)	Increased mortality risk for patients with MPM compared to SPM.

Kricker et al. 2013 [19]	2372/1206	13.56 versus 2.93 (hazard ratio)	Relative fatality risk was higher for a thick SPM than for a thick MPM.

Savoia et al. 2012 [4]	4938/270	65%/80% (OS)	Better overall survival for patients with MPM.

Bower et al. 2010 [5]	2506/48	80%/95.3% (OS)	Patients with MPM had better overall survival.

Ferrone et al. 2005 [3]	4484/385	15.8%/5.6% (MR)	Better prognosis for patients with MPM.

Doubrovsky and Menzies 2003 [14]	5250/298		Survival of patients with MPM is superior to survival of patients with SPM.

Slingluff Jr. et al. 1993 [25]	236/?	31%/25% (MR)	Not reduce survival for patients with MPM.
